# Inverse spin Hall effect in a complex ferromagnetic oxide heterostructure

**DOI:** 10.1038/srep28727

**Published:** 2016-06-27

**Authors:** Martin Wahler, Nico Homonnay, Tim Richter, Alexander Müller, Christian Eisenschmidt, Bodo Fuhrmann, Georg Schmidt

**Affiliations:** 1 Institut für Physik, Martin-Luther University Halle-Wittenberg, Halle, 06120, Germany; 2 Interdisziplinäres Zentrum für Materialwissenschaften, Martin-Luther University Halle-Wittenberg, Nanotechnikum Weinberg, Halle, 06120, Germany

## Abstract

We present spin pumping and inverse spin Hall effect (ISHE) in an epitaxial complex oxide heterostructure. Ferromagnetic La_0.7_Sr_0.3_MnO_3_ (LSMO) is used as a source of spin pumping while the spin sink exhibiting the ISHE consists of SrRuO_3_ (SRO). SRO is a ferromagnetic oxide with metallic conductivity, however, with a Curie temperature (*T*_*C*_) of 155 K, thus well below room temperature. This choice allows to perform the experiment above and below *T*_*C*_ of the SRO and to demonstrate that SRO not only shows an ISHE of a magnitude comparable to Pt (though with opposite sign) in its non magnetic state but also exhibits a finite ISHE even 50 K below *T*_*C*_.

The conversion of pure spin currents into charge currents has widely been investigated over the past decade and a large variety of materials have been used in these experiments^1^,^2^,^3^,^4^,^5^. In the prototypical setup a ferromagnet is excited to ferromagnetic resonance using a microwave signal. The resulting precession of the magnetization causes a spin current into an adjacent conducting material^6^. This spin current cannot be measured directly but first needs to be converted into a charge current or a voltage^7^,^8^. This so called spin-charge conversion can either be achieved by measuring spin-accumulation using secondary ferromagnetic contacts as spin selective voltage probes^9^ or using the ISHE^5^,^8^. Especially the ISHE has been of increasing importance over the last years. Reasons are not only the simplicity of the necessary experimental setup but also the possible application of SHE and ISHE in spintronics. The spin Hall effect can for example be used to increase the efficiency of spin transfer torque for MRAM application^10^ and the ISHE is not only an excellent candidate for measuring pure spin currents but is even under consideration for energy harvesting by converting spin currents generated by thermal gradients into electricity^11^. The materials which are used to measure the ISHE are plenty. Most of them are metals like Pt or Au^1^ and more recently W^12^ and Ta^5^,^10^thin films. But also for organic conductors^4^ and inorganic semiconductors^13^,^14^ISHE has been demonstrated. In view of the multitude of available complex oxides and the prospect of future oxide thin film based electronics^15^,^16^,^17^,^18^,^19^it is obvious that including a complex oxide into the catalogue of suitable materials would open up new perspectives. So far, however, only for sputtered indium tin oxide the ISHE was demonstrated by Qiu *et al*.^20^. Moreover it has been shown theoretically in 2012 that the ISHE can also appear in ferromagnetic materials^21^ a claim which has been followed by experimental proof in the subsequent years^22^,^23^. While it is difficult to setup a suitable experiment for ferromagnetic metals it is much easier to realize it in a complex oxide heterostructure. The fact that many ferromagnetic oxides have a Curie temperature below room temperature which is normally considered as a disadvantage can greatly facilitate the experiment, because it allows for switching the magnetization on and off just by changing the temperature, which is not an option for typical metallic ferromagnets where *T*_*C*_ is several hundreds of °C^24^. For our experiment we find the ideal candidate in the combination of LSMO and SRO, where both materials are ferromagnets at low temperature, however, above *T* = 155 K only the ferromagnetism of the LSMO prevails^25^.

## Results

**Figure 1:**
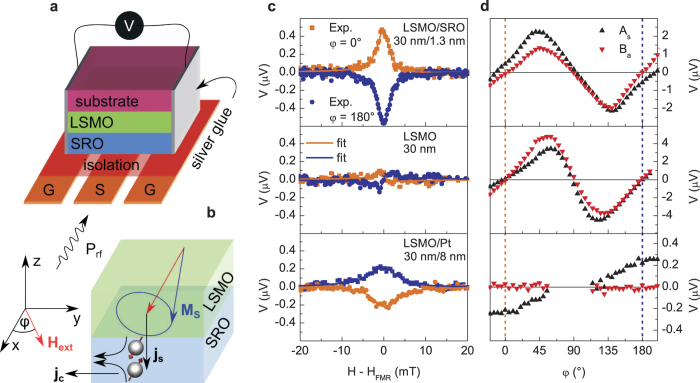
Measurement geometry for separating the ISHE voltage from the AMR-generated voltage. (**a**) Sample placed on waveguide separated by an electrically isolating coating. The generated voltage can be measured between the two contacts on the sample sides. (**b**) Depiction of the ISHE voltage generation. A spin current *j*_*s*_ generated by spin pumping from the LSMO into the SRO is converted to a charge current *j*_*c*_. (**c**) H-dependence of the generated voltage signal for two directions *φ* = 0 and *φ* = 180° for the three types of samples at a temperature of *T* = 190 K. For the LSMO/Pt and the LSMO/SRO samples a symmetric shape of the line is dominant, whereas for the pure LSMO sample, where only AMR contributes to the voltage signal, the symmetric component vanishes. Note that the voltage has opposite sign for SRO and Pt (**d**) Angular dependence of the symmetric and antisymmetric contribution from fits to *V*_*DC*_ = *A*_*s*_ × *L*_*s*_(*H*) + *B*_*a*_ × *L*_*a*_(*H*) + offset. At *φ* = 0 and *φ* = 180°, both symmetric and antisymmetric contribution become negligible. Nevertheless, for the LSMO/SRO and LSMO/Pt sample a clear symmetric contribution is present which does not contain any contribution from AMR.

In our experiments we use a total of nine samples all based on a (30 ± 0.4) nm LSMO layer on NdGaO_3_ substrate. Seven of these samples are covered by SRO. The layer thickness are 1.2 nm, 1.3 nm, 2.0 nm, 3.7 nm, 9.5 nm, 11.0 nm and 23.0 nm with error bars of ±0.4 nm and are named sample 1 to 7, respectively. The two remaining samples are used as reference samples, one without any cap layer on the LSMO and the other covered by (8 ± 1) nm of Pt. These two samples are referred to as reference 1 and reference 2, respectively. In a first set of measurements on sample 2 and reference 1 and 2 we determine the DC voltage over the sample during microwave excitation at a frequency of 9.6 GHz and a temperature of 190 K which is above T_*C*_ of SRO but below the T_*C*_ of LSMO (Fig. 1c). The magnetic field *μ*_0_*H*_ext_ is swept from below to above the resonance field for the LSMO and the measurement is repeated for different in-plane alignments of the magnetic field in steps of 5°. This set of measurements is performed on all three samples. For the analysis it is necessary to remove the contributions which stem from RF-rectification due to anisotropic magnetoresistance (AMR) which can occur in a conducting ferromagnet under RF excitation. For certain conditions the precessing magnetization can cause the resistance to oscillate in phase with the induced RF currents, leading to rectification and DC voltages^26^. Only when the external field is perpendicular to the RF field no rectification is expected. These AMR-caused voltages need to be carefully separated from the ISHE voltage in order to extract the right result as has been shown by Obstbaum *et al*.^27^. Furthermore, the microwave currents that are coupled inductively or capacitively to the conducting bilayer have to be considered^27^. These currents might cause an additional Oersted field. The effect can be particularly pronounced in lithographically defined structures where the waveguide has a thickness comparable to that of the bilayer. In our case, however, the waveguide is macroscopic and a conservative estimate shows that any contribution from this effect is several orders of magnitude below the ISHE that we observe.

As a first step we compare the voltage signal generated in the LSMO/SRO heterostructure (sample 2) with that of a single LSMO layer of the same thickness (reference 1). We fit the data by a lorentzian line shape with a symmetric and an antisymmetric part. According to Obstbaum *et al*.^27^ the ISHE voltage only results in a symmetric contribution, while the signal from AMR can result in both, symmetric and antisymmetric contributions. Figure 1d shows that for the single LSMO layer symmetric and antisymmetric contributions of finite amplitudes are generated at different magnetization directions in the sample plane. Only when the external magnetic field is directed along the waveguide (*φ* = 0 or *φ* = 180°), no DC voltage can be measured as is expected from theory^26^. We can thus conclude in reverse that for this alignment of the magnetic field any DC voltage generated in other LSMO based heterostructures does not stem from AMR rectification. It should be noted that both symmetric and antisymmetric contribution vanish at this particular angle because in the bilayer samples the vanishing antisymmetric contribution still shows that the angle is correctly aligned. Nevertheless, a possible misalignment of the angle might still lead to unwanted contributions from AMR. A simple estimate, however, shows that a misalignment of at least 10° would be necessary to provide an artifact of the observed magnitude while we can guarantee an alignment accuracy better than 1°. Asymmetries from the silver-glue electrodes can also be excluded because measurements with waveguides where the contacts are far away from the excitation region still show the same effect. Sample inhomogeneities are also negligible because the epitaxial quality of the layers is very high as has been shown by X-ray diffraction (see also sample fabrication).

For reference 2 (LSMO/Pt) the Pt has a much lower resistance than the LSMO and represents a short circuit for the AMR-generated signal while creating a strong ISHE voltage by itself. The angle dependent measurements (Fig. 1d) confirm that in this sample the maximum DC signal appears for the angle at which no AMR signal could be observed for reference sample 1, as expected from geometric considerations.

For LSMO/SRO heterostructures the result represents a mixture of the two because the SRO has much lower conductivity than Pt and thus a sizeable AMR contribution from the LSMO remains visible. For SRO the ISHE has the opposite sign when compared to Pt, which has also previously been observed for Mo^1^, Ta^10^ and W^12^.

Please note that in all measurements, a constant background has been subtracted which was independent from the direction and magnitude of the magnetic field.

For further analysis we limit ourselves to the amplitude of the symmetric contribution which can be precisely determined at the angle where the AMR signal and thus the antisymmetric contribution vanishes completely as we have shown for the pure LSMO layer (broken line in Fig. 1d). In this case, the spin Hall voltage can be written as


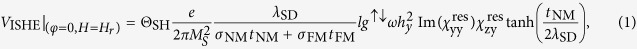


if out-of-plane excitation is neglected. The formula is derived as described in other publications^1^,^2^,^27^and contains the thicknesses *t*_FM_ and *t*_NM_ and conductivities *σ*_FM_ and *σ*_NM_ of LSMO and SRO, respectively, the saturation magnetization of the LSMO layer (*M*_S_), the length *l* over which excitation takes place, the excitation field in y-direction (*h*_*y*_), the excitation frequency (*ω*/2*π*) and the susceptibilities of the precessing magnetization at resonance (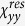 and 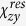).

**Figure 2:**
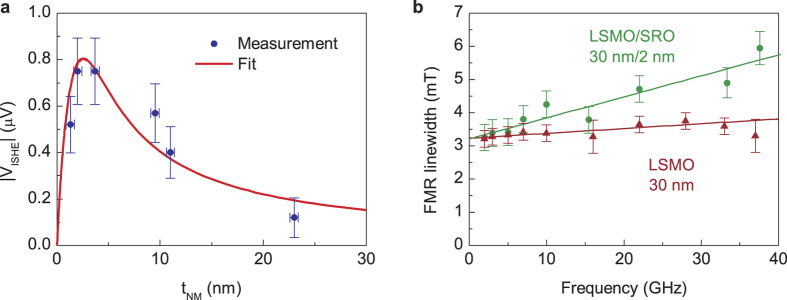
Quantitative analysis of the voltage generated by ISHE at *T* = 190 K. (**a**) ISHE voltage as a function of the paramagnetic SRO thickness with a fixed thickness of the ferromagnetic LSMO of 30 nm. From the fit to equation (2) a spin diffusion length of *λ*_SD_ = (1.5 ± 0.6) nm is obtained. (**b**) FMR linewidth as a function of resonance frequency. The damping parameters for the LSMO layer (reference 1) and one of the LSMO/SRO bilayers (sample 3) are determined from the slope. This data is used to calculate the spin mixing conductance of the LSMO/SRO interface. Note that a zero-frequency linewidth of more than 3 mT is present. This inhomogeneous line broadening is not associated with the investigated effect. It originates in material imperfections and does not influence our analysis as it is equal in both samples.

To determine the spin Hall angle Θ_SH_ of the SRO, the spin-diffusion length *λ*_*SD*_ of the SRO and the spin-mixing conductance *g*^↑↓^ of the LSMO/SRO interface must be determined. The latter is obtained by comparing the damping parameter *α* = 1.8 × 10^−3^ of the SRO-covered LSMO layer with that of the bare LSMO layer *α*_0_ = 4.1 × 10^−4^ similar to Azevedo *et al*.^2^. For this purpose FMR measurements are performed in the frequency range from 2 GHz to 37 GHz for both samples (Fig. 2b) and the damping parameters are extracted from the line widths. This yields a spin mixing conductance of *g*^↑↓^ = (1.1 ± 0.3) × 10^19^ m^−2^. To calculate the spin diffusion length *λ*_SD_ we measure the ISHE voltage on a total of seven samples with varying thickness of the SRO. The results are shown in Fig. 2a. These values are fit to


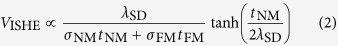


as suggested by Mosendz *et al*.^1^ resulting in *λ*_SD_ = (1.5 ± 0.6) nm. To determine the LSMO and SRO conductivities the conductivities of all bilayers are determined from 2-probe resistance measurements at a temperature at 190 K using the same leads as employed in the ISHE experiment as voltage probes. We use the relation *σ*_bilayer_(*t*_NM_ + *t*_FM_) = *σ*_NM_*t*_NM_ + *σ*_FM_*t*_FM_ to evaluate the conductivities. The conductivity of the LSMO is determined as *σ*_LSMO_ = (5.0 ± 0.8) × 10^4^ Ω^−1^ m^−1^ on a bare layer of LSMO and furthermore assumed as constant for all samples because all LSMO layers involved are grown with the same thickness and growth parameters. From the resistance of the bilayers the SRO conductivities are then calculated. Except for the bilayer with thinnest SRO layer (sample 1), the conductivity only slightly increases for the lower layer thicknesses but can be treated as constant within the error bars that are introduced due to the uncertainty of the layer thickness. For the questionable sample 1, we have to assume different material quality which may affect other physical properties. Therefore we exclude this sample from the analysis. The conductivity of the SRO is *σ*_SRO_ = (7.5 ± 1.1) × 10^5^ Ω^−1^ m^−1^. For the Pt sample we get *σ*_Pt_ = (1.8 ± 0.5) × 10^7^ Ω^−1^ m^−1^. In addition we use *M*_S_ = (3.8 ± 0.8) × 10^5^ A/m from SQUID-magnetometry, *l* = 600 *μ*m (width of the signal line), *h*_*y*_ = 20 A/m from DC-current approximation of the field generated by the input power of 40 mW, *ω*/2*π* = 9.6 GHz and the susceptibilities calculated from the ferromagnetic resonance and we obtain |Θ_SH_| = (0.027 ± 0.018) at *T* = 190 K with the sign being opposite to that of the spin Hall angle of Pt.

**Figure 3:**
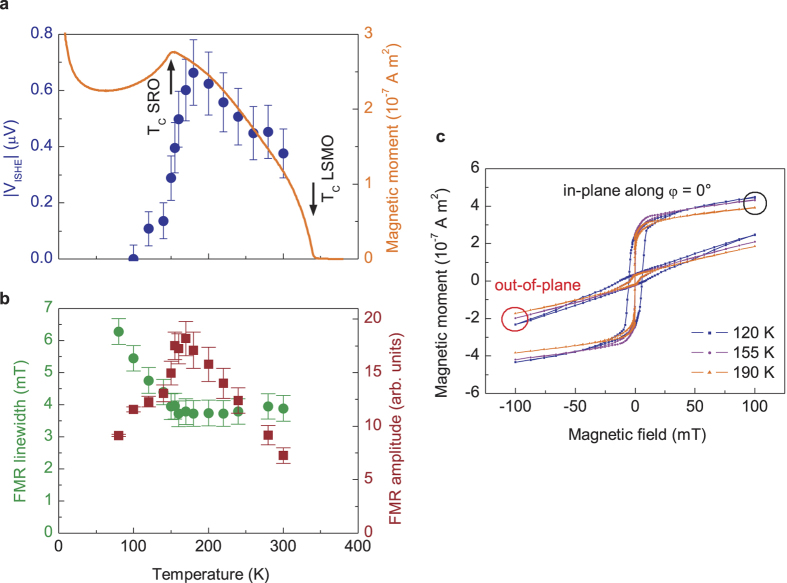
Temperature dependence. (**a**) Comparing the inverse spin Hall voltage with the magnetization of the bilayer (LSMO 30 nm/SRO 1.3 nm) measured in-plane at a constant field of 0.2 mT and subtracted by a constant contribution from the paramagnetic substrate. Below *T*_*C*_(SRO) the voltage generated due to ISHE strongly decreases but remains finite down to *T* = 100 K, where it is below the detection limit. Approaching *T*_*C*_(LSMO) the magnetization and therefore the spin Hall voltage decreases. (**b**) While obve *T*_*C*_(SRO) the FMR linewidth is constant, there is an increase below this temperature. The FMR amplitude still has a value of 50% of the maximum at 100 K. (**c**) Hysteresis loops measured for out-of-plane and in-plane direction at temperatures at *T*_*C*_(SRO) and 35 K below and above. The curves are corrected for the linear contribution of the strongly paramagnetic substrate. It should be noted that in the field range of ±100 mT the out-of-plane magnetization is not yet saturated.

In further experiments we measure the temperature dependence of the effect from 100 K to 300 K to investigate the influence of the ferromagnetism in SRO which has a Curie-temperature of 155 K. Figure 3a additionally shows the magnetization of the LSMO/SRO bilayer during cooldown in a field of 0.2 mT measured by SQUID magnetometry. The curve is typical for LSMO/SRO because the SRO tends to couple antiferromagnetically to the LSMO effectively reducing the total magnetization^28^. Figure 3c, however, shows hysteresis loops at different temperatures and for taken either in-plane or perpendicular to plane. Although these measurements show that the total in-plane magnetization of the LSMO barely changes when SRO is below *T*_*C*_, it cannot be concluded that the direction of magnetization remains constant in the applied magnetic field. A deviation in angle of 10° or less would barely show up in the measurement because it only determines the projection of the magnetization on the magnetic field. It can, however, be concluded that no additional out-of-plane magnetization appears as might be expected for single SRO layers^25^. In addition, a large increase in coercive field (both in- and out-of-plane) occurs when the SRO becomes ferromagnetic indicating a coupling between the two layers. It should be noted that the saturation magnetization of SRO is much smaller than that of LSMO (1.4 *μ*_*B*_/Ru^25^ and 3.7 *μ*_*B*_/Mn^29^ low temperature saturation magnetization for bulk material) which in addition with the much smaller layer thickness leads to the fact that the SQUID measurement mainly shows the magnetization of LSMO. Together with the cooling curve of the magnetization also the ISHE voltage is plotted for different temperatures. Obviously the ISHE voltage exhibits a maximum at around *T* = 180 K. When the temperature is increased starting from *T* = 180 K we observe a decay of the ISHE voltage. This is easily understood from the reduction of the magnetization (and thus the spin polarization) of the LSMO layer which also reduces the spin pumping.

Upon cooling beyond *T*_*C*_(SRO) the ISHE signal slowly disappears, however, it is noteworthy that a sizeable effect persists until well below *T*_*C*_(SRO) confirming other experiments showing ISHE in a ferromagnetic layer^22^. Note that the vanishing ISHE signal does not even prove that the ISHE itself is truly absent at lower temperatures. In principle it is possible either that due to its strong crystalline anisotropy the SRO may couple to the LSMO and pull the magnetization away from the magnetic field, thus changing the geometry of the experiment or that the magnetization vector of the SRO is non-collinear to that of the LSMO and the spin polarization of the current induced by the spin pumping is changed by spin transfer torque. A decreasing FMR amplitude and an increase in linewidth below *T*_*C*_(SRO) (Fig. 3b) point towards the first effect, however, the second effect cannot be ruled out.

For sake of clarity also possible artifacts from thermoelectric effects due to heating of the sample on resonance should be discussed. In principle the spin heat conveyer effect^30^ can create a temperature gradient which can result in a thermovoltage that reverses sign when the magnetic field is reversed. In LSMO, however, the magnons are short lived and do not extend far enough to create the signals that we observe. Theoretically also the Nernst-Ettingshausen effect^31^ can create a similar signal. Compared to our measurements it would, however, neither show a similar dependence on temperature nor layer thickness of the SRO so it can effectively be ruled out while in the pure LSMO layer no signal is observed at all, excluding the anomalous Nernst effect^32^.

In summary we have shown that SRO can act as a spin sink in a fully epitaxial heterostructure consisting of two complex oxide ferromagnets. SRO exhibits ISHE with a sign opposite to that of Pt. The spin Hall angle is smaller than for Pt and the effect persists even below the Curie temperature of the SRO. The fact that the effect can be investigated directly at the phase transition opens up new possibilities for future investigation of the ISHE.

## Methods

### Sample preparation

**Figure 4:**
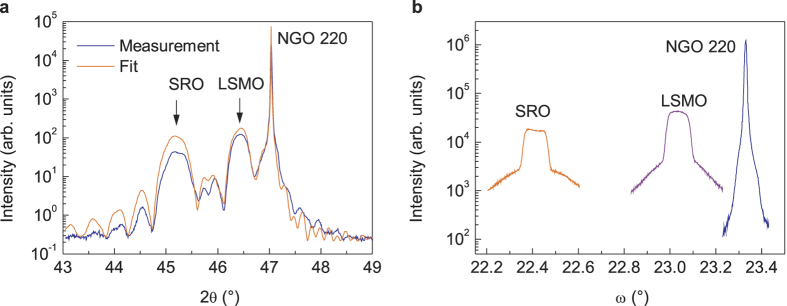
XRD charakterisation. (**a**) Omega-2Θ-scan of the 220-reflection of sample 7 (LSMO 30 nm/SRO 23 nm). The fit is in excellent agreement with the measurement and reproduces the thickness-fringes indicating low interface roughness. (**b**) Rocking curves for the same sample reveal a FWHM of 0.01° for the substrate and 0.1° for the LSMO and SRO layers. This is more than has been reported for other PLD grown oxide layers of similar thickness^33^,^34^and indicates a maximum mosaicity of 0.1° in both LSMO and SRO, respectively.

Pulsed Laser Deposition (PLD) is used to deposit layers of La_0.7_Sr_0.3_MnO_3_ (LSMO) with a thickness of 30 nm on orthorhombic NdGaO_3_ (110) substrates (NGO). Subsequently without breaking the vacuum a layer of SrRuO_3_ (SRO) with a thickness of *t*_NM_ is grown. Both materials are deposited using an oxygen pressure of 0.2 mbar at a substrate temperature of 650 °C. The laser fluency is 2.5 J/cm^2^ at a repetition rate of 5 Hz. Using this recipe a total number of 7 samples is fabricated with different respective thickness *t*_NM_. For each sample the layer thickness is verified by X-ray reflectometry. Furthermore for the samples with thickest SRO layer XRD measurements are carried out which are shown in Fig. 4. The presence of thickness-fringes in the *ω*2*θ*-scan indicates a low interface roughness. Complementary rocking-curves of the 220-reflex for all layers show a small contribution from mosaicity of the LSMO and SRO layers.

In addition two reference samples are fabricated, one single LSMO layer and one sample where the SRO is replaced by 8 nm of Pt which is also deposited without breaking the vacuum by magnetron sputtering. The samples are cleaved to approximately 2 mm times 5 mm rectangles where the substrate [001] direction is oriented along the short sides. On these sides copper leads are attached using silver glue to serve as voltage probes for the ISHE.

### Measurement

Like shown in Fig. 1a samples are placed on top of a coplanar waveguide with a 600 *μ*m wide inner conductor and 100 *μ*m gap made of a 35 *μ*m thick Cu layer on hydrocarbon ceramic laminate. The waveguide is electrically isolated by an approximately 100 nm thick polyimide layer. The waveguide is placed in a cryostat which is in constant magnetic field *μ*_0_*H*_ext_ of a rotatable electromagnet. A microwave current is transmitted through the waveguide whose RF magnetic field excites ferromagnetic resonance and thus spin precession in the LSMO layer. As depicted in Fig. 1b the precessing magnetization causes spin pumping to the adjacent SRO layer where the ISHE leads to spin scattering and consequently in the generation of a DC voltage. The cryostat is liquid nitrogen cooled allowing for temperature dependent measurement in the range from 300 K down to 80 K. It is possible to modulate the RF amplitude and to use a lock-in amplifier to measure the ISHE or to apply a small AC magnetic field allowing for lock-in detection of the absorption and thus the ferromagnetic resonance.

### Data analysis

In analogue manner to Obstbaum *et al*.^27^, the data from individual voltage measurements are fit to *V*_*DC*_ = *A*_*s*_ × *L*_*s*_(*H*) + *B*_*a*_ × *L*_*a*_(*H*) + offset with *L*_*s*_(*H*) = Δ*H*^2^/((*H* − *H*_*r*_)^2^ + Δ*H*^2^) and *L*_*a*_(*H*) = (*H* − *H*_*r*_)Δ*H*/((*H* − *H*_*r*_)^2^ + Δ*H*^2^) to obtain the amplitudes for the symmetric and antisymmetric contribution of the voltage signal *A*_*s*_ and *B*_*a*_ respectively. For the case of *φ* = 0 or *φ* = 180°, *A*_*s*_ is equal to *V*_ISHE_ since any contribution from AMR becomes zero for this configuration. FMR measurements have been carried out subsequently to the voltage measurements from which the susceptibilities at resonance can be extracted.

## Additional Information

**How to cite this article**: Wahler, M. *et al*. Inverse spin Hall effect in a complex ferromagnetic oxide heterostructure. *Sci. Rep*. **6**, 28727; doi: 10.1038/srep28727 (2016).
